# Supplementary material for: Giant lungfish genome elucidates the conquest of land by vertebrates
Source: Nature. 2021 Jan 18;590(7845):284–9. doi: 10.1038/s41586-021-03198-8 (PMC7875771; doi:10.1038/s41586-021-03198-8)
Supplement: Supplementary file 1 — This file contains Supplementary Methods, Supplementary Results, Supplementary Table legends, and Supplementary References. [file 41586_2021_3198_MOESM1_ESM.pdf]

nature portfolio

https://doi.org/10.1038/s41586-021-03198-8

Supplementary information

Giant lungfish genome elucidates the conquest of land by vertebrates

In the format provided by the
authors and unedited

SI Guide

Supplementary Information

Giant Lungfish genome elucidates the conquest of land by vertebrates – Supplementary File 1

Axel Meyer[1*¶#], Siegfried Schloissnig[2*], Paolo Franchini[1*], Kang Du[3,4*], Joost Woltering[1*], Iker Irisarri[5,11], Wai Yee Wong[6], Sergej Nowoshilow[2], Susanne Kneitz[7], Akane Kawaguchi[2], Andrej Fabrizius[8], Peiwen Xiong[1], Corentin Dechaud[9] Herman Spaink[10], Jean-Nicola Volff[9], Oleg Simakov[6¶], Thorsten Burmester[8¶], Elly M. Tanaka[2¶#], Manfred Schartl[3, 4¶#]

[1]Department of Biology, University of Konstanz, Germany.
[2]Research Institute of Molecular Pathology (IMP), Campus-Vienna-Biocenter, Vienna, Austria.
[3]Developmental Biochemistry, Biocenter, University of Würzburg, Germany.
[4]The *Xiphophorus* Genetic Stock Center, Texas State University, San Marcos, TX, USA.
[5]Department of Biodiversity and Evolutionary Biology, Museo Nacional de Ciencias Naturales (MNCN-CSIC), Madrid, Spain.
[6]Department of Neuroscience and Developmental Biology, University of Vienna, Austria.
[7]Biochemistry and Cell Biology, Biocenter, University of Würzburg, Germany.
[8]Institut für Zoologie, Universität Hamburg, Germany.
[9]Institut de Genomique Fonctionnelle, Ecole Normale Superieure, Universite Claude Bernard, Lyon, France.
[10]Faculty of Science, Universiteit Leiden, The Netherlands.
[11]Current address: Department of Applied Bioinformatics, Institute for Microbiology and Genetics, University of Goettingen, and Campus Institute Data Science, Goettingen, Germany

* co-first authors
¶ supervising authors
# co-corresponding authors: axel.meyer@uni-konstanz.de; oleg.simakov@univie.ac.at; thorsten.burmester@uni-hamburg.de; elly.tanaka@imp.ac.at; phch1@biozentrum.uni-wuerzburg.de

Table of Contents

	Page
**Supplementary Methods**	3
Generation of Hi-C library	3
Genome assembly: in depth methods and work flow	4
Assembly validation	6
Hi-C based scaffolding methodology and workflow	7
Genome assembly correction	9
Annotation of non-protein coding genes	9
Non-coding RNA annotation	9
miRNA target sites prediction	10
Length comparison of 5' UTRs, 3' UTRs, and introns	10
Molecular clock analyses	10
In situ hybridization	11
Annotation of *Hox* clusters	11
Hox gene RNAseq analysis	12
Limb enhancer analysis	12
**Supplementary Results**	13
Assessment of assembly quality	13
Heterozygosity and Q-value of the lungfish genome assembly	14
Positively selected genes	14
Intron size comparisons	14
Differential accumulation of transposable elements in genic regions	15
Localized expansion of Conserved Non-Coding elements	15
Accelerated evolution of Conserved Non-Coding elements	15
Limb enhancer analysis	16
**Supplementary Tables**	17
**References**	20

Supplementary Methods

Generation of Hi-C library

Hi-C library was generated as described in Nagano *et al.*[1] and Wutz *et al.*[2], with the following modifications. 0.2 g of snap-frozen lungfish spleen was powdered and fixed with 1% formaldehyde at RT for 10 min. The fixation was stopped by adding ice-cold glycine (125 mM f. c.). Fixed cells were strained with 100 µm cell strainer to generate single cells, collected by centrifugation (200 x g for 10 min at 4°C), and washed with ice-cold PBS twice (200 x g for 10 min at 4°C). 1×10^6 cells were collected and incubated in ice-cold lysis buffer (10 mM Tris–HCl pH 8, 10 mM NaCl, 0.2% Igepal CA-630, 1% Triton-X100, protease inhibitor cocktail EDTA free) for 30 min on ice. Nuclei were pelleted by centrifugation (250 x g, 10 min at 4°C), washed once with 1.25x NEBuffer 3 and resuspended in 1.25x NEBuffer3. SDS was added (0.6% f. c.) incubated (950 rpm, 2 h at 37°C). Triton X-100 was added for quenching the SDS (3.3 % f. c.) and the nuclei were incubated (950 rpm, 2 h at 37°C). Restriction digest with DnpII (in 1x DnpII buffer from NEB; 2,000 U per 0.25 million cells) was performed overnight (950 rpm at 37°C). Using biotin-14-dATP, dCTP, dGTP, and dTTP, the DnpII restriction sites were filled in with Klenow for 1 h at 37°C. Ligation (T4 DNA ligase) was performed overnight at 18°C. De-crosslinking was carried with proteinase K in SDS buffer overnight at 65°C. The biotinylated DNA was purified by sequential phenol/chloroform extractions and EtOH precipitation. 20 µg of biotinylated DNA was used for library preparation. Excess biotin was removed with T4 DNA polymerase (30 min at 37°C), EDTA was added to stop the reaction (10 mM f. c.), then The biotinylated DNA was sheared by the Covaris S2 (fragment size: around 400bp, duty factor: 10%; peak incident power: 5 W; cycles per burst: 200; time: 60 sec). After end repair (T4 DNA polymerase, T4 DNA PNK, Klenow in the presence of dNTPs in T4 DNA ligation buffer; for 30 min at RT), the DNA was purified. A double-size selection using DNA purification beads. Biotinylated ligation products were isolated using MyOne Streptavidin C1 Dynabeads (Life Technologies) following the manufacture protocol. dA-tailing was carried on beads: dATP was added with Klenow exo- (for 1h at 37°C), then the enzyme was heat-inactivated (20 min at 65°C). After two washes in binding buffer and one wash in T4 DNA ligation buffer, Illumina Tru-seq adapter (Index #10_ TAGCTT) was ligated onto Hi-C ligation products bound to streptavidin beads in T4 DNA ligase. After two washes, the captured biotinylated DNA was amplified with Q5 Ultra PCR mix (NEB).

Genome assembly: in depth methods and work flow

Broken alignments, due to two reads having a low-quality region where the alignment passes through, are detected and repaired (LAstitch). The approximate error of the reads is estimated by inspecting the level of identity achieved in the alignments to it (LAq). This error rate is then used to trim the reads (LAq). The repeats are detected based on coverage statistics (LArepeat). Transitive transfer is used to homogenize the repeat annotation across the read-mass and ensure that repeats smaller than the minimum alignment length at the beginning and end of a read are annotated as well (TKhomogenize). This repeat annotation is then combined with the repeat masks used when aligning the reads initially (TKcombine). Larger regions in the reads not spanned by any read are detected and resolved, these regions are referred to as gaps (LAgap). Resolving in this context entails selecting a group of overlaps from one of the sides of the gap and discarding the other. This then necessitates another round of trimming of the reads (LAq). The remaining alignments are then filtered to remove local and repeat-induced alignments (LAfilter). After this, only true overlaps should remain, which are then used to build the assembly graph (OGbuild). Optionally, at this stage the reads used for constructing the contigs, as selected during assembly graph touring (OGtour), can be corrected (LAcorrect) and used for constructing the contigs (tour2fasta). The sequence of command corresponding to this workflow is given below.

```
# commands prefixed with [B] are executed for each read block

# PLACEHOLDERS:
# - {READ_BLOCK} - a single read block, e.g. LFISH.1, LFISH.321
# - {ALL_READ_BLOCKS} - all read blocks, e.g. LFISH.1, LFISH.2, … LFISH.n
# - {LFISH_DB}, {LFISH_FIX_DB}, {LFISH_FIX_CORR_DB} - path to the database, e.g. LFISH.db
# - {READ_BLOCK_NUMBER} - always used jointly with a {READ_BLOCK},
#   used to indicate+ the number after the database name,
#   e.g. {READ_BLOCK} = LFISH.4 then {READ_BLOCK_NUMBER} = 4

# compute all alignments for a random subset of database blocks
# derive a coarse repeat annotation to mask the worst repeat elements
# transfer the annotation to all other reads based on the alignments

[B] daligner -A -t20 -T4 {READ_BLOCK} {ALL_READ_BLOCKS}
[B] LAmerge -n 16 {LFISH_DB} {READ_BLOCK}.las {READ_BLOCK_DIRECTORY}
[B] LArepeat -h 4.0 -l 3.5 -c 30 -t repeats_init -b {READ_BLOCK_NUMBER} {LFISH_DB} {READ_BLOCK}.las
[B] TKhomogenize -I {READ_BLOCK_NUMBER}.repeats_init -I hrepeats -b {READ_BLOCK_NUMBER} {LFISH_DB}
{READ_BLOCK}.las

# after a repeat annotation for a random subset has been derived and
# transfered to every other reads merge the resulting annotations
# {READ_BLOCK_x_NUMBER} refers to the {READ_BLOCK_NUMBER} of each block used
# in the TKhomogenize calls above
TKcombine {LFISH_DB} hrepeats_init {READ_BLOCK_x_NUMBER}.hrepeats_init …

# with the repeat annotation at hand we then perform the alignments
# for all read block comparisons

[B] daligner -mhrepeats_init -t20 -T4 {READ_BLOCK_x} {ALL_READ_BLOCKS}
[B] LAmerge -n 16 {LFISH_DB} {READ_BLOCK}.las {READ_BLOCK}

# derive read-quality quality information from the alignments
[B] LAq -b {REAd_BLOCK_NUMBER} {LFISH_DB} {READ_BLOCK}.las
```

```
# merge the block specific quality information
TKmerge -d {LFISH_DB} q

# repair sequencing artifacts and low quality regions in the reads
[B] LAfix -g -1 {LFISH_DB} {READ_BLOCK}.las {READ_BLOCK}.fasta

# create new read database
FA2db -x 4000 -c source {LFISH_FIX_DB} *.fasta
DBsplit -s 400 {LFISH_FIX_DB}

# repeat what has been done above for the initial repeat masking

[B] daligner -A -t20 -T4 {READ_BLOCK} {ALL_READ_BLOCKS}
[B] LAmerge -n 16 {LFISH_FIX_DB} {READ_BLOCK}.las {READ_BLOCK}
[B] LArepeat -h 4.0 -l 3.5 -c 30 -t repeats_init -b {READ_BLOCK_NUMBER} {LFISH_FIX_DB} {READ_BLOCK}.las
[B] TKhomogenize -I {READ_BLOCK_NUMBER}.repeats_init -I hrepeats -b {READ_BLOCK_NUMBER} {LFISH_FIX_DB}
{READ_BLOCK}.las

# after a repeat annotation for a random subset has been derived and
# transfered to every other reads merge the resulting annotations
# {READ_BLOCK_x_NUMBER} refers to the {READ_BLOCK_NUMBER} of each block used
# in the TKhomogenize calls above

TKcombine {LFISH_FIX_DB} hrepeats_init {READ_BLOCK_x_NUMBER}.hrepeats_init …

# with the repeat annotation at hand we then perform the alignments
# for all read block comparisons

[B] daligner -mhrepeats_init -t20 -T4 {READ_BLOCK_x} {ALL_READ_BLOCKS}
[B] LAmerge -n 16 {LFISH_FIX_DB} {READ_BLOCK}.las {READ_BLOCK_DIRECTORY}

# repair broken alignments
[B] LAstitch {LFISH_FIX_DB} {READ_BLOCK}.las {READ_BLOCK}.stitch.las

# compute read qualities and trimming information
[B] LAq -b {READ_BLOCK_NUMBER} {LFISH_FIX_DB} {READ_BLOCK}.stitch.las

# merge quality and trim information
TKmerge -d {LFISH_FIX_DB} trim
TKmerge -d {LFISH_FIX_DB} q

# compute repeat annotation
[B] LArepeat -c 30 -t repeats -b {READ_BLOCK_NUMBER} {LFISH_FIX_DB} {READ_BLOCK}.stitch.las

# merge repeat annotation
TKmerge -d {LFISH_FIX_DB} repeats

# transfer the annotation
[B] TKhomogenize -I repeats -b {READ_BLOCK_NUMBER} {LFISH_FIX_DB} {READ_BLOCK}

# combine it with the initial repeat annotation
TKcombine {LFISH_FIX_DB} hrepeats #.hrepeats
TKcombine {LFISH_FIX_DB} repeats_all hrepeats repeats

# resolve haplotypic breaks and breaks due to leftover weak regions in the reads
[B] LAgap -m 1 -L -t trim {LFISH_FIX_DB} {READ_BLOCK}.stitch.las {READ_BLOCK}.gap.las

# update trimming information
[B] LAq -b {READ_BLOCK_NUMBER} -u -t trim -T trim2 {LFISH_FIX_DB} {READ_BLOCK}.gap.las

# merge trim information
TKmerge -d {LFISH_FIX_DB} trim2

# filter alignments
[B] LAfilter -p -n 300 -t trim2 -u 0 -T -r repeats_all {LFISH_FIX_DB} {READ_BLOCK}.gap.las
{READ_BLOCK}.filter.las

# merge filtered alignments
LAmerge -n 16 {LFISH_FIX_DB} merged.filter.las *.filter.las

# create overlap graph
OGbuild -t trim2 {LFISH_FIX_DB} merged.filter.las assembly.graphml

# tour the overlap graph
OGtour.py -c {LFISH_FIX_DB} assembly.graphml
```

```
# correct all reads used for contig construction
LAcorrect -j 4 -r assembly.rids -b {READ_BLOCK_NUMBER} {LFISH_FIX_DB} merged.filter.las
merged.filter.corrected

# create database with the final reads
FA2db -c source -c postrace {LFISH_FIX_CORR_DB} *.corrected.fasta

# output contigs
tour2fasta.py -c {LFISH_FIX_CORR_DB} -t trim2 {LFISH_FIX_DB} assembly.tour.graphml
```

MARVEL has a modular design, which in essence is a tool-kit that allows fine control over each step of the assembly process which is necessary to adapt the assembly procedure for highly repetitive genomes. Other assemblers that treat repetitive sequences as comprehensively as MARVEL have run-time issues on such large genomes while other long-read assemblers are not as adjustable to gain accurate assembly of highly repetitive regions. Another characteristic feature for the long-read assembly part is the read-patching procedure which uses other reads to patch-correct stretches of artefacts and maintains read length rather than cutting the reads at those regions as many other assemblers do.

This modular design allowed us to perform a full comparison of a fraction of the reads against all the others. Thereby enabling us to derive the location of the most abundant repeat classes with these 1% of the reads. Furthermore, we then used the alignments generated in the process to annotate the same repeat elements in all the reads used within the assembly.

For the current MARVEL source code repository see https://github.com/schloi/MARVEL and for sample execution scripts see https://github.com/schloi/MARVEL/examples/.

Assembly validation

To gauge repeat and segmental collapse we implemented an approach to SDA to identify regions of unusually high coverage. In brief, we mapped the ONT reads to the assembly using winnowmap2 and identified regions where the coverage exceeds 3 standard deviations from the mean (Extended Data Fig. 10). We detected 2248 such regions comprising about 34,405,000 bp (0.099% of the combined scaffolds length). Only 9.3% out of the 0.099% portion of the scaffolds that show high coverage are not associated with annotated repeats, meaning that only 0.0092% of the assembly might be subject to the caveats of repeat and segmental collapse (Supplementary Table 14).

Furthermore, we validated the assembly and scaffolding in comparison to the chromosome-scale meiotic scaffolding from ref.[3] and is available as described in ref.[4].

Hi-C based scaffolding methodology and workflow

The high level of repetitiveness of large genomes poses a challenge for HiC based scaffolding. Contact depletion in repetitive regions coupled with sequence biases disturbing the assumed uniform spread of digest sites throughout the genome result in biases that lead to suboptimal scaffolding. We developed an agglomerative hierarchical clustering based scaffolding approach utilizing various normalization techniques to overcome these hurdles while also achieving high performance (Extended Data Fig.1).

The scaffolder can be used in two ways. Fully automated scaffolding of a list of contigs and their associated contacts. Guided scaffolding, which allows forcing a global structure onto a scaffold, e.g. reusing an existing scaffolding, but still allowing for local reordering of the contigs. This can be used for a final polishing pass on the scaffolds or after contigs have been assigned to an existing scaffold and placed close to their assumed position in the scaffold.

We created initial clusters by selecting the largest contigs with the fewest contacts between them. Each contig serving as a single cluster. We then added contigs based on unique assignability to the clusters. This was followed by scaffolding the cluster separately, visual inspection of an approximate contact map derived during the scaffolding process and return of wrongly assigned contigs to the set of unassigned contigs. We created contact maps for all clusters (effectively a while genome contact map) and merged or split clusters based on the signal within those. The process of assigning contigs, scaffolding, merging and splitting clusters was repeated until no more useful changes could be made to the clusters.

Normalization encompasses contact repositioning, length normalization and midpoint computation. Contact repositioning entails the adjustment of the contact positions to the nearest digest site. For length normalization we employ two strategies resulting in two measures for the effective sequence length. One dividing the sequence in equally sized bins, with the counts of the bins corresponding to the number of contacts contained in them. The effective sequence lengths correspond to the number of non-zero bins multiplied by the bin size. The other measure uses the number of active digest sites (i.e. sites present in the sequence with an actual contact mapping to it) as a proxy for sequence length. The midpoint, i.e. the middle of the contig, is used to determine the orientation (reverse complement or not) in relation to other contigs. Here contact depletion can effectively result in the breakdown of the assumption that the midpoint corresponds to the length divided by two. To illustrate, imagine a contig with left half having no repetitive elements, whereas the right half does. Most contacts will therefore be in the left

half. If the midpoint is not adjusted, this would result in the contig preferentially being attached to others at its left side.

The high-level structure of the scaffolding algorithm is as follows:

Pre-processing

a. Reading of contig sequence lengths from provided fasta or fasta index
b. Reading of the location of the digest sites
c. Reading of the contacts and remapping to the closest digest site
d. Effective sequence length computation based on binning
e. Midpoint computation
f. Effective length computation based on digest sites
g. Build initial scaffold graph (node = contigs, edges = contacts)

Scaffolding (repeated until no more nodes can be joined)

a. Node scoring – for each node compute the most likely node to the left and right
b. Nodes having each other as most likely neighboring nodes are joined into paths. Paths effectively represent a node in the graph and subsume their contained nodes.
c. Compute effective lengths and midpoints for new nodes (ie. paths)

Post-processing

a. Write final set of paths (ie. scaffolds)

The scaffolding itself creates a graph structure from the contact data and contigs contained in a cluster. Turning contigs into nodes and contacts into edges. Based on the edges, the most likely left and right neighbour of each node were computed and continuous chains of nodes having each other as their most likely neighbour merged into paths (effectively a new node) subsuming their constituent nodes. This process was repeated until no more paths can be constructed (Extended Data Fig.1a). The final Hi-C contact map of the scaffolded lungfish genome assembly is displayed in Extended Data Fig.1d). The correspondence of the estimated chromosome DNA content and of DNA content of the largest scaffolds is given in Supplementary Table 15.

Several aspects of the MARVEL HiC scaffolder are notable. First, the algorithm runs very quickly, so it was possible to go rapidly go through scaffolding attempts and evaluate their quality, even on such a large genome. These iterations allowed us to learn that the axolotl Bionano scaffolds were not ideal for HiC scaffolding. Second, a normalization procedure for calculating the contact frequencies between contigs was developed. This is very important in

the landscape where most of the genome consists of contact-depleted repetitive sequence, which has a strong effect on contact frequencies. Third, we developed a misjoin evaluation tool to detect and correct misjoins.

HiC data can not only be used to scaffold genomes but also to validate the structural soundness of a given scaffold and by extension the integrity of a contigs above a certain length threshold. For this purpose, we developed an experimental misjoin detection tool that looks for structurally abnormal contact arrangements within contigs and used it in order to screen the assembled contigs. This resulted in a putative list of 1583 misjoins, indicating a fairly low number of errors in the assembly of such a large highly repetitive genome.

Genome assembly correction

For correction of the assembly a two-step procedure was used. First, the DNAseq reads were checked for contamination by human, mouse, Drosophila, axolotl, *C. elegans*, and a compilation of bacterial genomes obtained from NCBI using FastQ Screen[5] and subsequently mapped to the genome using bowtie2[6]run in the sensitive mode (--very-sensitive). We used the default parameters for the alignment score threshold, which is -90 for the given read length, and would allow the reads with up to 16 mismatches per 150bp to still be aligned. The overall mapping rate was roughly 88%. Examination of the distribution of scores showed that 55% of the mapped reads mapped with 0-3 mismatches per 150 bp. The mapped reads were used to correct indels, gaps, SNPs, and small local misalignments using Pilon[7].

In the second step, 14 RNAseq samples (499,838,488 reads) used for the transcriptome assembly (second and third set of reads described in the "Transcriptome assembly" section) were used to further improve the error correction in the transcribed regions. The reads were mapped to the reference corrected in step 1 using HISAT2[8] run with the option –dta with the default alignment score threshold of -30 which roughly corresponds to up to 6 mismatches per 150 bp. The overall average alignment rate was 84%, of which 91% mapped with up to 3 mismatches per 150 bp. Finally, the mapped reads were used to correct indels and gaps only using Pilon.

Annotation of non-protein coding genes

Non-coding RNA annotation. Non-protein coding genes were annotated using the following procedure: 1) Transfer RNA (tRNA) were predicted with the program tRNAscan-SE v2.0.3[9] with a score threshold of 65 using the GtRNAdb v2.0 June release database[10]; 2) the other classes of non-coding RNAs (ribosomal RNAs, small cytoplasmatic RNA, microRNA

precursors and long intergenic non-coding RNAs) were inferred by aligning genomic sequences against the Rfam v14.1 January release database[11] using the program Infernal v1.1.2[12] with an E-value cutoff of 1e-6. Alignments results from the two approaches were translated in “gff3” format using custom Perl scripts. The same procedure was applied to the genomes of the nine other focal species.

miRNA target sites prediction. The sequences of mature miRNAs extracted from miRBase v22[13] were used to predict the miRNA target sites in eight focal species with annotation of 3’ UTRs. First, the nonredundant sequences of mature miRNAs conserved in both aquatic (pisces) and terrestrial (other vertebrata) vertebrates were extracted. Then the miRNA target sites on the 3’ UTRs of canonical mRNAs were predicted using miRanda v3.3[14] with parameter “-strict -en 20”, which requires the perfect matching on the seed region and the minimum free energy of the binding is lower than 20 kcal/mol.

Length comparison of 5’ UTRs, 3’ UTRs, and introns. As there is no annotation of 5’ UTRs and 3’ UTRs in the genomes of *X. laevis* and *A. mexicanum*, these two genomes were excluded in this comparison. In the other eight focal genomes, longest mRNA for each gene was selected as canonical mRNA that represents the gene. The length of 5’ UTR, 3’ UTR, and intron of the canonical mRNAs was compared. In addition, the comparison was performed for the 697 one-to-one orthologous genes.

Molecular clock analyses

Divergence times were inferred with a relaxed molecular clock with autocorrelated rates, as implemented in MCMCTree within the PAML package v.4.9h[15]. A total of six fossil calibrations were used to calibrate the following nodes with uniform priors[16]: (i) the root at 465-475 Ma, (ii) the gar-zebrafish split at 249-299 Ma, (iii) the lungfish-tetrapod split at 420-475 Ma, (iv) the tetrapod crown group at 335-365 Ma, the axolotl-xenopus split at 249-290 Ma, and the common ancestor of human, green anole, and chicken at 318-365 Ma. We used approximate likelihood calculations based on the gradient and Hessian matrix of the likelihood at the ML estimates of branch lengths, calculated with CODEML (within the PAML package) under the best-fit JTT+Γ4 model. Priors on ancestral rate “rgene_gamma” were set to G(2, 7.797), corresponding to a diffuse prior with mean rates of 0.2565 amino acid replacements $site^{-1}$ Myr^{-1}. Mean rates were approximated using the average root-to-tip paths in the PhyloBayes tree and a mean root age of 470 (mean between maximum-minimum bounds). The prior on the σ2 parameter (“sigma2_gamma”) was set to G(2,2) indicating substantial among-lineage rate heterogeneity. The tree prior assumed a uniform birth-death process with default parameters.

The time unit was set to 100 Myr. Two independent MCMC chains were run for twenty million cycles, sampling every 1,000, after the initial 20,000 cycles that were discarded as burnin. Convergence, was checked *a posteriori* in Tracer v.1.5 and all parameters obtained high ESS values >200

In situ hybridization

In situ hybridization was performed as described[17,18]. All oligonucleotide sequences are given in Supplementary Table 15. Modifications were implemented for lungfish *sall1* and axolotl *hoxd9*. Probe preparation for axolotl *hoxd9* and lungfish *sall1* was performed with a different procedure. 120bp oligos spanning 600 bp for axolotl *hoxd9* and 1200bp for lungfish *sall1* were ordered together with a T7 promoter tailed reverse primer for each long oligo. Double stranded fragments were generated by combining 1µl of 10µM FW and RV oligo in a 25 µl PCR reaction (containing 0.5 µl dNTP, 5ul 5x HF reaction buffer and 0.5 µl Phusion HF polymerase M0539 NEB, adjusted with H_2O to 25 µl) with one cycle reaction 98°C 2min, 50°C 10min, 72°C 10min. Correct synthesis of double stranded DNA at equal efficienciesamongst reactions for the same gene was confirmed using 3% agarose gel analysis. Subsequently reaction for the same gene were pooled and column purified. 600ng of purified PCR product was subsequently used for a probe synthesis reaction using T7 polymerase and DIG labeled nucleotides. Probes were purified using the standard protocol. In situ hybridisation was carried out for these genes at 65°C instead of 68°C because of the short length of the probe.

Annotation of *Hox* clusters

Hox genes were first identified by BLAST against an embryonic transcriptome derived from stage 52 pectoral fins, posterior trunk and caudal fin[18] using vertebrate orthologs as queries. BLAST against the *Neoceratodus* genome identified the contigs containing the four *Hox* clusters:

hoxa1-hoxa14 - NFORS_037472_pilon_pilon;
hoxb1-hoxb10 - NFORS_046705_pilon_pilon;
hoxb13 - NFORS_040414_pilon_pilon;
hoxc1 - *hoxc13* - NFORS_041640_pilon_pilon;
hoxd1 - *hoxd12* - NFORS_044832_pilon_pilon;
hoxd13 - NFORS_035197_pilon_pilon.

Open reading frames (ORFs) were annotated using transcripts identified from the transcriptome dataset. Whenever only a partial transcript was identified in the transcriptome the annotation

was completed using homology with the coelacanth or published sequences from *Neoceratodus* (*hoxa14*)[19].

<u>*Hox* gene RNAseq analysis</u>

The hox gene RNAseq analysis was performed on a previously deposited RNAseq dataset obtained from and right pectoral fins of a st. 52 lungfish larva (SRR6297462-SRR6297470)[18]. *Hox* expression values were determined by mapping the filtered reads to the coding sequences as obtained from the genome. The final FPKT values (Fragment per Kilobase of transcript per Thousand mapped reads) presented in Extended Data 8c were derived similar to calculations normally used for the FPKM. FPKM was not used because of the low number of total reads mapping because mapping was not done genome wide but only against the *Hox* coding regions. RNAseq samples from anterior, middle and posterior fin cuts together representing the entire pectoral fin - were grouped. The reads for each fin were further analysed together. Extended Data 8c shows the mean between left and right fins with the error bars indicating the difference between the highest and the lowest sample.

<u>Limb enhancer analysis</u>

The human sequence for 330 non-redundant enhancer elements showing limb expression in enhancer Vista[20] were extracted and BLAST-searched against the genomes of *Xenopus laevis* (XENLA9_2), *Xenopus tropicalis* (XENTR_10.0), *Nanorana parkeri* (nanPar1), axolotl (AmexG_v3.0.0), reedfish (ErpCal1.1), sterlet (ARUT1.2), gar (LepOcu1), elephant shark (Callorhinchus_milii-6.1.3), coelacanth (LatCha1), and *Neoceratodus*. BLAST hits were considered conserved for conservation over 100bp of length. Enhancers sequences were considered conserved ancestral to bony fish if shared between human (and/or elephant shark, and/or reedfish, and/or sterlet, and/or gar). Enhancers sequences were considered new to sarcopterygians if shared between human (and/or *Neoceratodus* and/or coelacanth), but absent from (elephant shark, and reedfish, and sterlet, and gar). Enhancer sequences were considered new to tetrapods if shared between humand (and/or *Nanorana parkeri*, and/or *Xenopus tropicalis*, and/or *Xenopus laevis*, and/or axolotl), but absent from (Elephant shark, and reedfish, and sterlet, and gar, and *Neoceratodus*, and coelacanth).

Supplementary Results

Assessment of assembly quality

As one way of assessing the quality of genome contigs and scaffolds, we examined the alignment of the *de novo* assembled transcriptome contigs to both genome contigs and genome scaffolds.

260,478 out of 283,820 contigs aligned to the genome contigs using GMAP (REF: https://doi.org/10.1093/bioinformatics/bti310), while 253,781 align to the scaffolds. This small differential is due to genome contigs that were not included in the scaffolds. We checked the size distribution of these 23,342 transcripts that did not map to the genome and over 18,000 of them are 300 bp or shorter. This left 5,000 unmapped transcripts greater than 300 bp in size (~1.8%). Among these, we find hits in the bacterial database and so these transcripts are very likely contamination from bacteria and other flora/fauna that could not be removed from the tissue before RNA preparation.

Extended Data Fig. 2b-e show the proportion of the transcript sequence covered by the alignment to the contigs (Extended Data Fig.2b) and scaffolds (Extended Data Fig.2c) on the X-axis and the number of transcripts that have this coverage on the Y-axis. Most of the transcripts align fully to both contigs and scaffolds as can be seen from the large peak around 100% in both figures. Transcripts that only aligned partly to contigs, but aligned either completely or to a greater extent to the scaffolds are of particular interest. Extended Data Fig.2d-e show the distribution of 17,549 transcripts that had partial alignments to the contigs and improved alignment to the scaffolds. These plots show that the scaffolding shifted the coverage of many transcripts to 100%, validating the accuracy of the scaffolding.

As a final assessment, we specifically focused on roughly 4400 transcripts that showed 40-60% alignment to the contigs. Of those approximately 1208 did not show marked improvement in alignment to the scaffolds. We wanted to determine whether this might be due to scaffolding problems, or due to artifactual contigs in the de novo assembled transcriptome – a well-known problem. When we examined these 1208 further, only 478 were longer than 500 bp, and therefore the majority of those 1208 sequences most probably represented unidentifiable bits and pieces that are known to plague de novo transcriptome assemblies. We examined the longest transcript contig (10kb) in this class, and extensive BLAST alignment of this transcript and its sub-parts to NR showed signs of a mis-assembled transcript contig, another hallmark of de novo transcriptome assemblies. Although we cannot state that all genome contigs were correctly

placed, we conclude from this analysis that the genome scaffolds show very good accuracy in ordering the genome contigs.

Heterozygosity and Q-value of the lungfish genome assembly

The heterozygosity was estimated to be 0.0965-0.1278 % using genomescope (http://genomescope.org/).

The Q-value was computed using meryl (https://github.com/marbl/meryl) and merqury (https://doi.org/10.1101/2020.03.15.992941), In brief, Illumina reads used for correction were processed with Trimmomatic[21], low quality bases were trimmed, and adapters removed. We concurrently processed batches of 10M reads to count k-mers (k=23) with meryl, merged the results and calculated the Q-value with merqury.

Based on this procedure we estimate a Q-value of 26 for the lungfish. For comparison, the Q-value for the axolotl assembly is 30. This difference most likely being attributable to the increase in repeat content, which poses a challenge for correction, over the axolotl.

Positively selected genes

In the lungfish lineage 158 genes are under positive selection (Supplementary Table 9). Positively selected genes included a wide spectrum of cellular and molecular functions and pathways with no particular enrichment except for genes expressed in the liver. In the common lineage of lungfish and tetrapods a total of 259 genes show signatures of positive selection, many of which are related to estrogen and female reproduction related categories (Supplementary Table 9).

Intron size comparisons

Several studies have suggested that the size of intragenic non-coding sequences and the extent of intron expansion is not random but is associated with organismal features such as metabolic rate[20] or functional categories of genes[22], e.g., developmental vs. non-developmental genes. Thus, we compared intron sizes between developmental genes and non-developmental genes in the lungfish genome using Mann-Whitney U test. The list of developmental gene was obtained from a previous study[22]. Intron sizes for all genes were extracted from the genome annotation gff file. We found that in the lungfish genome, the size of introns in developmental genes is significantly smaller than that of non-developmental genes ($p= 2.166e-08$, Mann-Whitney U test) (Supplementary table 8) and similar to what was observed in the axolotl genome[22].

Differential accumulation of transposable elements in genic regions

To study the contribution of TEs in gene expansion (mainly introns), we calculated the proportion of different classes of TEs within genes (Supplementary Table 17). In lungfish, axolotl and caecilian, the proportion of LTRs showed similar increase in longer genes (Extended Data Fig. 6b). Further analysis of LTR family composition in genes showed that LTR/DIRS are significantly enriched in genic regions (43.9%) compared to an average of 15.1% of other LTR classes and concentrate in introns (0% - 4.5% in exons; 4.1% - 44.2% in introns) (Extended Data Fig. 6c, d). It is tempting to speculate that the permissiveness of the selection/drift regime[23] to maintain vastly expanded introns in both lungfish and axolotl made the accumulation of longer LTR elements possible in such transcribed regions.

Localized expansion of Conserved Non-Coding elements

To identify regulatory elements in the Australian lungfish lineage, we relied on whole-genome alignments to retrieve a set of conserved non-coding elements (CNEs) (see Supplementary Information). The conserved non-coding element (CNE) dataset inferred from the multispecies whole-genome alignment was used to check in which portions the lungfish genome expanded disproportionally (See Supplementary Information).

Based on 223 intergenic CNE pairs identified from human, chicken, axolotl and lungfish genomes, we noticed, as expected, that these genomic regions expanded substantially more compared to other tetrapods with relatively small genomes (i.e. human and chicken). Interestingly, while as similar level of expansion characterizes axolotl and lungfish the genome expansion in *Neoceratodus* seems to be more localized (Supplementary Table 18).

Accelerated evolution of Conserved Non-Coding elements

The total 17,070 identified CNEs were searched for lungfish-accelerated regions, defined as non-coding genomic elements that are highly conserved in the focal species set, but strikingly diverged in the lungfish lineage. This approach allowed us to identify 308 genomic regions with signatures of accelerated evolution in lungfish (Supplementary Table 19). We intersected the accelerated lungfish CNEs with a set of experimentally validated human and mouse noncoding fragments with gene enhancer activity, as assessed in transgenic mice (data from "VISTA Enhancer Browser"[20]). We found that our accelerated CNEs intersected two enhancers: "hs2142", flanking a Plasminogen activator (PLAU) and a Vinculin gene (VCL) gene, with expression domains in forebrain, heart, hindbrain, midbrain and somite; "hs607", flanking a

Glutathione S-transferases (MGST1) and a LIM domain (LMO3) gene, with expression domains in hindbrain and neural tube.

Limb enhancer analysis

The fins of sarcopterygian, or lobe- finned, fishes display an evolutionary trend towards elaboration of the endochondral skeleton. This has resulted in their namesake sturdy lobe-like fins with extensive distal branching that are suitable for substrate-based locomotion. The terrestrially adapted tetrapod limb can be interpreted as an extreme example of this trend. The availability of a high-quality lungfish genome allowed us to investigate the evolutionary history of the regulatory architecture of sarcopterygian fins and limbs. First, the presence of 330 human and mouse non-redundant validated limb enhancers from Enhancer Vista[20] was assessed across gnathostome genomes (see Supplementary Methods). While 113 of these enhancers show conservation across bony fish, we found evidence for a sarcopterygian origin for 31 and only nine appear specific for tetrapods (Fig. 4a). Altogether, this shows ancient conservation of the regulatory architecture of fins and limbs and is consistent with the presence of a hand-like domain in *Neoceratodus*[18]. Seven of the sarcopterygian specific enhancers are related to genes whose expression can be directly linked to the evolution of the sturdy, elongated and distally branched skeleton that characterizes fleshly-lobed fins (Extended Data Fig.8b**)**. *Sox5*[24], *prrx1*[25] and *BMPR1b* (*BMPreceptor1b*)[26] each influence the length of the limb skeleton. *Gli3* is involved in the anterior-posterior patterning of the limb and could be involved in the evolution of the sarcopterygian mono-basal fin type[27,28] *Sall1* is involved in the distal branching of the autopod[29]. The autopodial -like expression of *sall1* in the lungfish fin (Fig. 4b) is in accordance with the sarcopterygian gain of enhancer *hs72* that drives LacZ expression in the mouse limb (Fig. 4b). Since no such *sall1* domain is present in zebrafish pectoral fins[30], the gain of *sall1* fin expression in the sarcopterygian lineage likely contributed to the evolution of the branching sarcopterygian lobed-fins as present in lungfish and tetrapods, and is also observed in extinct species such as *Sauripterus*. Whereas s*all1* appears to have a role in fin bud induction in zebrafish[31], we find it to be transiently expressed in ray-finned fins with a posterior bias. This contrasts with its expression during lungfish fin development where *sall1* is present in the complete endochondral autopod-like domain we identified before[32] resembling the tetrapod expression in the autopod.

Supplementary Tables

All Supplementary Tables are available on Mendeley Data (doi:…………..)

Supplementary Table 1. Basic statistics for the lungfish genome long-read sequencing and final assembly.

Supplementary Table 2. Assessment of the completeness of the genome assembly after annotation. The orthology search pipeline BUSCO was used with the Core Vertebrate Genes (CVG) and Vertebrata conserved genes (vertebrata_odb9) gene sets.

Supplementary Table 3. Comparison of numbers and structural features of different non-coding RNA classes and regions in lungfish and other vertebrates. The spreadsheet has the following sections:

1) Number of different types of ncRNAs in ten focal genomes. The lungfish genome contains 17,095 ncRNA genes, including 1,042 tRNA genes, 1,771 rRNA genes, and 3,974 microRNA genes. Length of 5' UTR, 3' UTR, CDS, and introns of canonical mRNAs.
2) Predicted miRNA target sites in eight genomes. Compared to other species, lungfish does not show significant difference in miRNA target density, suggesting the potentially neutral evolution of 3' UTR in lungfish.
3) Length of 5' UTR, 3' UTR, CDS, and intron in eight focal genomes. Lungfish has longer non-coding regions in the genes than other species.

Supplementary Table 4. Lungfish repetitive element statistics after the first round of masking. The table reports the repetitive element, number of elements, length (bp) occupied in the whole genome, percentage of sequence (%), average_copy_length (bp).

Supplementary Table 5. TE statistics after double masking, with merged with results from the first round of masking. The table reports the repetitive element, number of elements, length (bp) occupied in the whole genome, percentage of sequence (%), average_copy_length (bp).

Supplementary Table 6. Classification of consensus sequences from RepeatModeler by DeepTE, PASTEC and blast. The table shows the further classification result of each repetitive element consensus sequence from other annotators. "NA" refers to no matching result from the tool. The column "merge_strategy" suggests the best way to merge annotations from different tools.

Supplementary Table 7. Repertoire of the small non-coding RNA processing machinery genes in vertebrates. Presence or absence of genes were taken from ref.[33] and data of Australian lungfish and axolotl added. Presence (green) or absence (red) is indicated.

Supplementary Table 8. Comparison of intron sizes (in bp) between developmental and non-developmental genes in the lungfish genome

Supplementary Table 9. List of genes under positive selection in model 1 and model 2 and functional clustering. The spreadsheet has the following sections:

1) Positively selected genes in the lungfish genome (model 1) and in the common lineage of lungfish and tetrapods (model2).
2) Functional clustering by the DAVID and Ingenuitiy software for genes identified for model 1.

3) Functional clustering by the DAVID and Ingenuitiy software for genes identified for model 2.

Supplementary Table 10. Numbers of gene families that are significantly expanded or contracted in lungfish and other vertebrates. Results are from analyses using the CAFE program, version 4. Numbers are given for each branch of the phylogeny depicted in Figure 1.

Supplementary Table 11. Gene family dynamics in lungfish and other vertebrates. The spreadsheet has the following sections:

1) Gene families that were significantly ($p<0.01$) expanded or contracted in the lungfish branch
2) Gene families that were significantly ($p<0.01$) expanded or contracted on ancestral and terminal branches in 10 vertebrate species.

Supplementary Table 12. Number of functional pulmonary surfactant genes compared among species. The gene number is the sum of intact and truncated predictions.

Supplementary Table 13. Repertoire of olfactory and taste receptors. The number of functional olfactory receptors and taste receptor genes are given for lungfish and nine representative aquatic, amphibian or terrestrial species. Numbers are the sum of intact and truncated predictions. Odorant receptor are assigned to groups relating to the origin of the respective odors according to ref.[34].

Supplementary Table 14. Number and length of the regions in the genome that are not annotated as repetitive by RepeatMasker but having a coverage in excess of 3 standard deviants.

Supplementary Table 15. Rank order list of estimated chromosome DNA content and of DNA content in scaffolds. Left column: List of estimated chromosome DNA content. Chromosomal DNA content was calculated by measuring chromosome area from Rock et al 1996 and determining the fraction of the total with a genome size of 43 Gb. Right column: List of DNA content in scaffolds. This list is ordered by size and does not imply any relationship to the chromosomes listed on the left.

Supplementary Table 16. List of Oligonucleotides. This table list the oligonucleotide sequences used for in-situ hybridization probe synthesis for axolotl *hoxd9*, *hoxc13*, *hoxd9*, lungfish *sal1*, *hoxc13*, *sox9*, and cichlid *hoxc13*.

Supplementary Table 17. Counts of repetitive elements in genic regions. The table reports the genomic features (i.e. intron and exon), subfeatures (i.e. UTR, intron/exon number), repetitive element classes, number of elements (bp), length of features (bp), percentage of feature occupied by repetitive element (%) and numerical order of subfeatures (used to generate the plot).

Supplementary Table 18. Distance between CNE pairs in human, chicken, axolotl and lungfish. The table reports the 223 pairs of non-exonic conserved elements (CNE) that were identified in lungfish and three tetrapods (human, chicken and axolotl), and used to calculate the intergenic distance and the region-specific expansion of the lungfish genome. The selected informative CNE pairs 1) were present in the four species genomes, 2) were located in intergenic space of the same contig/chromosome in each species and 3) did not have a gene in between them. Mean and median expansion in comparison to axolotl and lungfish (the lineage that have undergone drastic genome expansion) are shown.

Supplementary Table 19. CNE showing accelerated evolution in lungfish. The program phyloP was used to test the non-coding conserved elements (CNE) for lineage-specific accelerated evolution in the lungfish lineage, using as complementary tree the other nine lineages in our multispecies alignment. The p-value for each CNE was computed using a likelihood ratio test using the "ACC" mode implemented in phyloP and corrected with the Benjamini–Hochberg false discovery rate (FDR) multiple test correction procedure. CNE ID, size and location in the human genome chromosome are shown. The last column indicates whether the focal CNE is located in intergenic or in genic space (UTR or intron).

References

1. Nagano, T. *et al.* Comparison of Hi-C results using in-solution versus in-nucleus ligation. *Genome Biol.* **16**, 175 (2015).
2. Wutz, G. *et al.* Topologically associating domains and chromatin loops depend on cohesin and are regulated by CTCF, WAPL, and PDS5 proteins. *EMBO J.* **36**, 3573–3599 (2017).
3. Smith, J. J. *et al.* A chromosome-scale assembly of the axolotl genome. *Genome Res* **29**, 317–324 (2019).
4. Nowoshilow, S. & Tanaka, E. M. Introducing www.axolotl-omics.org – an integrated -omics data portal for the axolotl research community. *Exp. Cell Res.* **394**, 112143 (2020).
5. Wingett, S. W. & Andrews, S. FastQ Screen: A tool for multi-genome mapping and quality control. *F1000Res* **7**, 1338 (2018).
6. Langmead, B. & Salzberg, S. L. Fast gapped-read alignment with Bowtie 2. *Nat. Meth.* **9**, 357–359 (2012).
7. Walker, B. J. *et al.* Pilon: An integrated tool for comprehensive microbial variant detection and genome assembly improvement. *PLoS ONE* **9**, e112963 (2014).
8. Kim, D., Langmead, B. & Salzberg, S. L. HISAT: A fast spliced aligner with low memory requirements. *Nat. Meth.* **12**, 357–360 (2015).
9. Chan, P. P. & Lowe, T. M. tRNAscan-SE: Searching for tRNA genes in genomic sequences. *Methods Mol. Biol.* **1962**, 1–14 (2019).
10. Chan, P. P. & Lowe, T. M. GtRNAdb 2.0: An expanded database of transfer RNA genes identified in complete and draft genomes. *Nucleic Acids Res.* **44**, D184-189 (2016).
11. Kalvari, I. *et al.* Rfam 13.0: shifting to a genome-centric resource for non-coding RNA families. *Nucleic Acids Res.* **46**, D335–D342 (2018).
12. Nawrocki, E. P. & Eddy, S. R. Infernal 1.1: 100-fold faster RNA homology searches. *Bioinformatics* **29**, 2933–2935 (2013).
13. Kozomara, A., Birgaoanu, M. & Griffiths-Jones, S. miRBase: from microRNA sequences to function. *Nucleic Acids Res* **47**, D155–D162 (2019).
14. Enright, A. J. *et al.* MicroRNA targets in *Drosophila*. *Genome Biol.* **5**, R1 (2003).
15. Yang, Z. PAML 4: Phylogenetic Analysis by Maximum Likelihood. *Mol. Biol. Evol.* **24**, 1586–1591 (2007).
16. Marjanović, D. Recalibrating the transcriptomic timetree of jawed vertebrates. bioRxiv (2019) https://doi.org/10.1101/2019.12.19.882829.
17. Woltering, J. M. *et al.* Axial patterning in snakes and caecilians: evidence for an alternative interpretation of the Hox code. *Dev. Biol.* **332**, 82–89 (2009).

18. Woltering, J. M. *et al.* Sarcopterygian fin ontogeny elucidates the origin of hands with digits. *Sci. Adv.* **6**, eabc3510 (2020).
19. Feiner, N., Ericsson, R., Meyer, A. & Kuraku, S. Revisiting the origin of the vertebrate Hox14 by including its relict sarcopterygian members. *J. Exp. Zool. Part B Mol. Dev. Evol.* **316**, 515–525 (2011).
20. Visel, A., Minovitsky, S., Dubchak, I. & Pennacchio, L. A. VISTA Enhancer Browser--a database of tissue-specific human enhancers. *Nucleic Acids Res.* **35**, D88-92 (2007).
21. Bolger, A. M., Lohse, M. & Usadel, B. Trimmomatic: A flexible trimmer for Illumina sequence data. *Bioinformatics* **30**, 2114–2120 (2014).
22. Nowoshilow, S. *et al.* The axolotl genome and the evolution of key tissue formation regulators. *Nature* **554**, 50–55 (2018).
23. MacManes, M. D. The Oyster River Protocol: a multi-assembler and kmer approach for de novo transcriptome assembly. *PeerJ* **6**, e5428 (2018).
24. Smits, P. *et al.* The transcription factors *L-Sox5* and *Sox6* are essential for cartilage formation. *Dev. Cell* **1**, 277–290 (2001).
25. Cretekos, C. J. *et al.* Regulatory divergence modifies limb length between mammals. *Genes Dev.* **22**, 141–151 (2008).
26. Demirhan, O. *et al.* A homozygous BMPR1B mutation causes a new subtype of acromesomelic chondrodysplasia with genital anomalies. *J. Med. Genet.* **42**, 314–317 (2005).
27. Tanaka, M. Fins into limbs: Autopod acquisition and anterior elements reduction by modifying gene networks involving 5'*Hox*, *Gli3*, and *Shh*. *Dev. Biol.* **413**, 1–7 (2016).
28. Onimaru, K. *et al.* A shift in anterior–posterior positional information underlies the fin-to-limb evolution. *eLife* **4**, e07048 (2015).
29. Kawakami, Y. *et al.* Sall genes regulate region-specific morphogenesis in the mouse limb by modulating Hox activities. *Development* **136**, 585 (2009).
30. Camp, E., Hope, R., Kortschak, R. D., Cox, T. C. & Lardelli, M. Expression of three spalt (sal) gene homologues in zebrafish embryos. *Dev. Genes Evol.* **213**, 35–43 (2003).
31. Harvey, S. A. & Logan M. P. O. *sall4* acts downstream of *tbx5* and is required for pectoral fin outgrowth. *Development* **133**, 1165–1173 (2006).
32. Woltering, J. M. *et al.* Sarcopterygian fin ontogeny elucidates the origin of hands with digits. *Sci. Adv.* **6**, eabc3510 (2020).

33. Biscotti, M. A. *et al.* The small noncoding RNA processing machinery of two living fossil species, lungfish and coelacanth, gives new insights into the evolution of the argonaute protein family. *Genome Biol. Evol.* **9**, 438–453 (2017).
34. Niimura, Y. Olfactory receptor multigene family in vertebrates: from the viewpoint of evolutionary genomics. *Curr. Genomics* **13**, 103–114 (2012).
