# Supplementary material for: Giant lungfish genome elucidates the conquest of land by vertebrates
Source: Nature. 2021 Jan 18;590(7845):284–9. doi: 10.1038/s41586-021-03198-8 (PMC7875771; doi:10.1038/s41586-021-03198-8)
Supplement: Supplementary file 4 — Basic statistics for the lungfish genome long-read sequencing and final assembly. [file 41586_2021_3198_MOESM4_ESM.docx]

**Sequencing Statistics**

|  | **reads** | **bases** | **N50** | **average** |
| --- | --- | --- | --- | --- |
| FutureGenomics | 69.951.665 | 601.709.513.637 | 9.150 | 8.602 |
| Nextomics UL | 1.572.689 | 49.354.762.645 | 44.502 | 31.382 |
| Nextomics | 24.710.996 | 532.445.021.346 | 27.451 | 21.547 |

**Assembly Statistics**

| bp in Contigs | 36.526.164.435 bp |
| --- | --- |
| Largest Contig | 14.781.449 bp |
| Contig N50 | 1.835.606 bp |
| Scaffolds | 39 |
| bp in Scaffolds | 34.557.648.077 bp |
| Largest Scaffold | 4.922.309.470 bp |
| Scaffold N50 | 1.751.869.518 bp |
| Contigs in Scaffolds | 48.505 |
